# Intestinal Haemorrhage and Colitis Induced by Treatment With Osimertinib for Non-Small-Cell Lung Carcinoma: A Case Report

**DOI:** 10.3389/fphar.2022.854277

**Published:** 2022-04-05

**Authors:** Wang Shujun, Lou Lili, Yang Lei, Wang Feng, Zhan Hefeng

**Affiliations:** ^1^ Department of Gastroenterology, The First Affiliated Hospital of Zhengzhou University, Zhengzhou, China; ^2^ Department of Respiratory and Critical Care Medicine, The First Affiliated Hospital of Zhengzhou University, Zhengzhou, China; ^3^ Department of Endoscopy Center, The First Affiliated Hospital of Zhengzhou University, Zhengzhou, China; ^4^ Department of Pathology, The First Affiliated Hospital of Zhengzhou University, Zhengzhou, China; ^5^ Department of Radiology, The First Affiliated Hospital of Zhengzhou University, Zhengzhou, China

**Keywords:** intestinal haemorrhage, drug-induced colitis, osimertinib, epidermal growth factor receptor tyrosine kinase inhibitor, aumolertinib, Non-small-cell lung carcinoma (NSCLC)

## Abstract

**Background:** Osimertinib is recommended either as the first-line therapy for sensitizing EGFR-mutations (FLAURA trial) or at progression to first-/second-generation EGFR inhibitors in the presence of resistance mutation T790M (AURA 3 study). It can effectively improve the prognosis of patients with NSCLC with manageable adverse reactions. Among adverse events, intestinal haemorrhage is rare and requires extensive study on its potential lethality.

**Case presentation:** A 59-year-old female, diagnosed with relapsed stage IV (cT4N2M1c) NSCLC with T790M mutation of the EGFR gene, received osimertinib treatment. Eight months after osimertinib treatment, she complained of lower abdominal pain and haematochezia without haemorrhoids. Potential causes of intestinal haemorrhage other than osimertinib toxicity were ruled out. Colonoscopy examination showed severe colitis with grade 3 CTCAE. Osimertinib was discontinued, and prednisone 0.5 mg/kg was administered. Follow-up endoscopy showed no pathological findings. A novel third-generation EGFR-TKI, aumolertinib, was administrated. Five months after aumolertinib initiation, CT evaluation showed stable disease (SD), and this patient was free of colitis recurrence.

**Conclusion:** To our knowledge, this is the first case report of severe colitis as an adverse event associated with osimertinib. Although osimertinib is the standard treatment for NSCLC in patients with T790M mutation and has fewer side effects, colitis may occur after months of treatment. Aumolertinib, a novel third-generation EGFR-TKI, might be an effective alternative for the treatment of patients with NSCLC experiencing colitis from osimertinib.

## Introduction

Specific tyrosine-kinase inhibitors (TKIs) targeting genomic alterations are currently in clinical development and show impressive activity and survival improvement in lung carcinoma (Lamberti et al., 2020). Among the TKIs, osimertinib is a third-generation epidermal growth factor receptor tyrosine-kinase inhibitor (EGFR-TIK) that is recommended either as the first-line therapy for sensitizing EGFR-mutations [FLAURA trial (Soria et al., 2018) or at progression to first-/second-generation EGFR inhibitors in the presence of resistance mutation T790M (AURA 3 study]. It is more effective than traditional pemetrexed plus platinum therapy in patients with T790M-positive advanced NSCLC (Mok et al., 2017). Osimertinib is also better tolerated compared to other EGFR inhibitors (Cho et al., 2019). Although there are many studies reporting its effectiveness and safety, adverse events related to this drug treatment have also been reported, including transient asymptomatic pulmonary opacities that might misdiagnose mild ILD or tumor progression (Lee et al., 2018). The most common adverse events in patients with EGFR T790M -positive advanced NSCLC after approved treatment with EGFR-TKIs are diarrhoea and rash according to AURA trial (Yang et al., 2017). For gastrointestinal adverse effects, diarrhoea (Yi et al., 2019), nausea, and decreased appetite are the most common all-cause adverse events (Jänne et al., 2015). This is the first case report of intestinal haemorrhage following osimertinib treatment and description of a novel third-generation EGFR-TKI, aumolertinib, which has a promising effect in NSCLC as an alternative treatment for osimertinib in NSCLC patients with T790M mutation.

## Case Presentation

**FIGURE 1:**
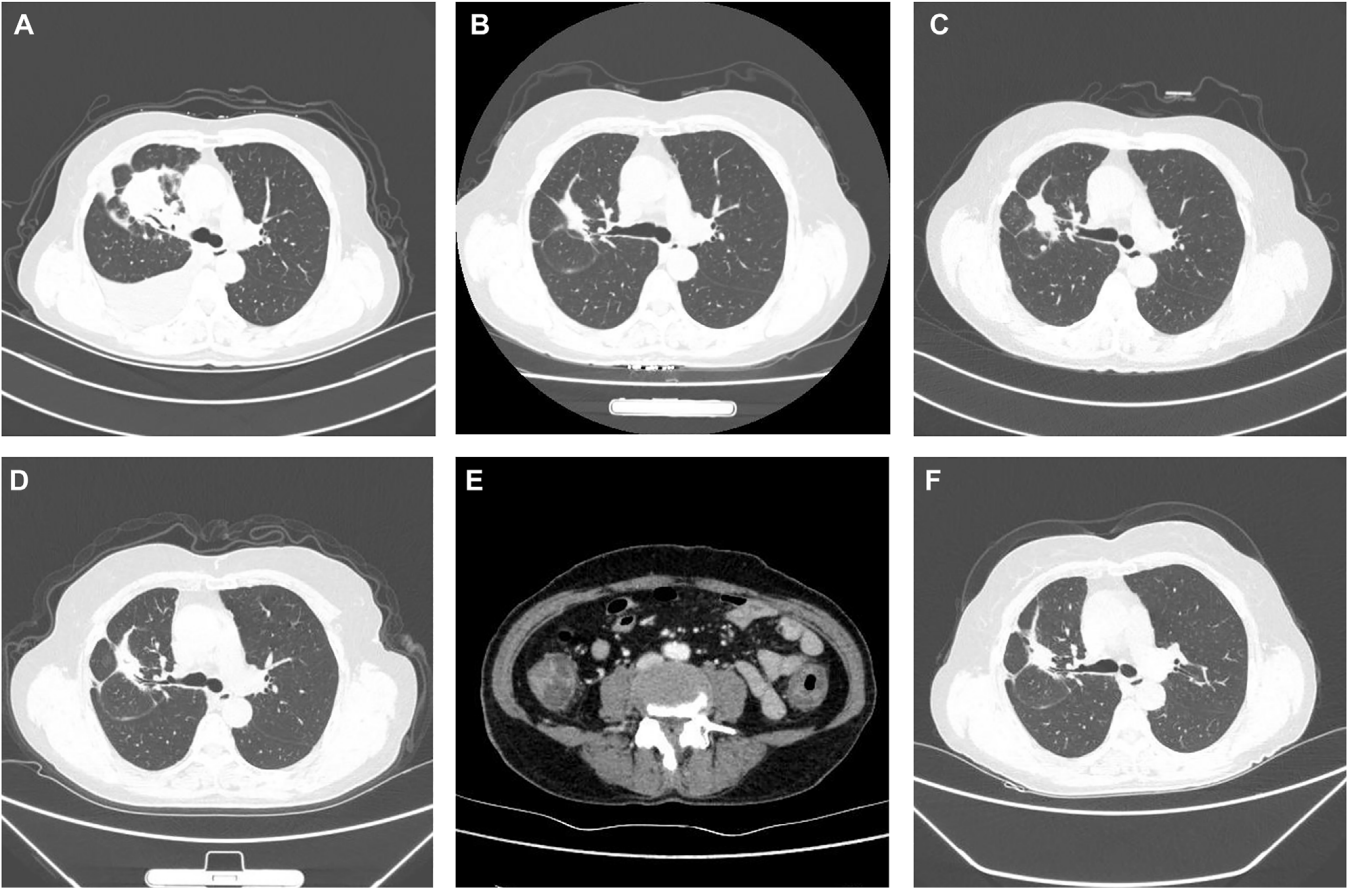
Clinical course according to CT scans. **(A)** Baseline CT scan at diagnosis in October 2019. **(B)** PR on icotinib in June 2020. **(C)** Disease progression in October 2020. **(D)** PR to osimertinib in January 2021. **(E)** Abdomen and pelvis CT in June 2021 showed that oedema had caused wall thickening of the colon. **(F)** SD on aumolertinib in November 2021.

A 59-year-old female non-smoker was initially diagnosed with clinical stage IV (cT4N2M1c, TNM classification seventh edition (Goldstraw et al., 2007) lung carcinoma with pleural and bone metastasis. She had no history of chronic obstructive pulmonary disease, hypertension, diabetes mellitus, colonial disease or haemorrhoids. Chest CT showed a lesion in the upper lobe of the right lung with enlarged lymph nodes and pleural effusion (Figure 1A). Magnetic resonance imaging (MRI) scans showed no metastases to the brain. Next-generation sequencing (NGS) examination suggested that tumours carrying exon 19 deletion mutations in the gene for EGFR might be sensitive to EGFR-TKI drugs. Therefore, the patient was treated with oral icotinib 125 mg three times daily.

After 8 months of drug therapy, a routine CT follow-up showed that lesion shrinkage was a partial response (PR) to treatment (Figure 1B). However, disease progression was observed 12 months after treatment. CT re-examination showed an enlarged mass (Figure 1C). First-generation EGFR-TKI drug resistance was considered. Subsequent NGS indicated an EGFR T790M mutation. Osimertinib 80 mg once daily was administered. She achieved PR 3 months after treatment with osimertinib (Figure 1D).

**FIGURE 2:**
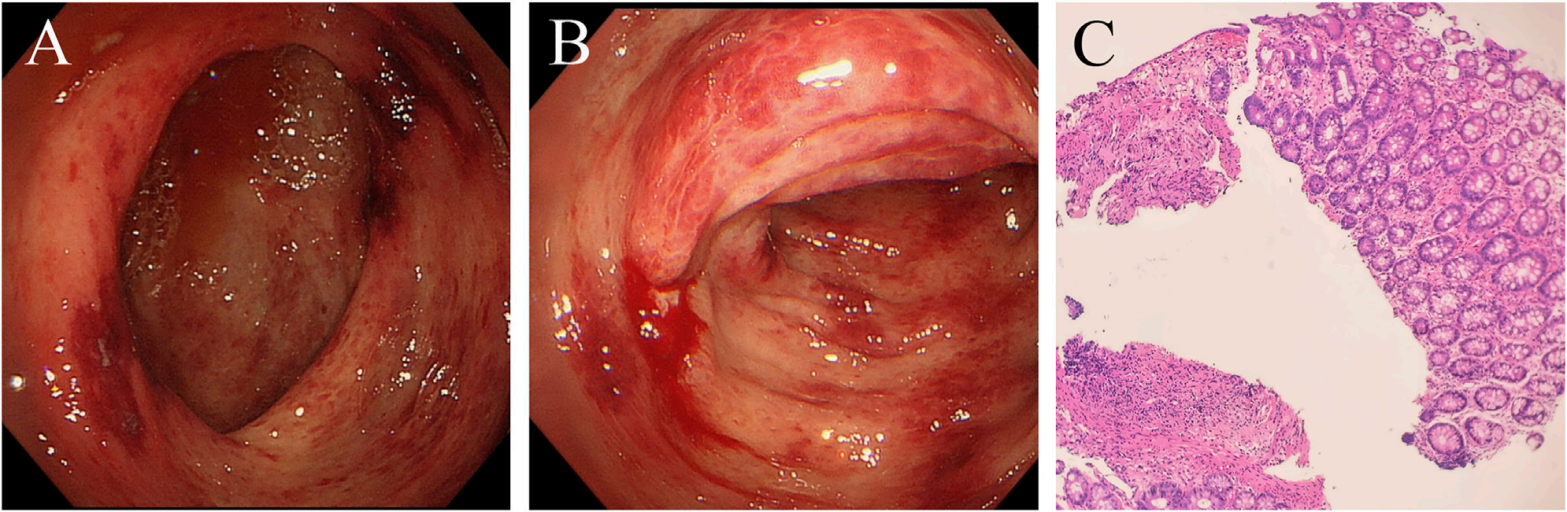
Colonoscopy shows oedema, erythema and bleeding ulcers from the ileocecal area to the sigmoid colon. Formation of the pseudomembrane and necrosis were not found. **(A)** ileocecal area. **(B)** transverse colon. **(C)** Pathological examination of the transverse colon. Mucosal ulcers are formed, and lamina propria inflammatory cells are infiltrated. Dilation of small blood vessels and vascular stasis were observed. The lamina propria also showed ischaemic change. (haematoxylin and eosin stain, x100).

However, 8 months after treatment with osimertinib, she complained of lower abdominal pain and had five to eight bloody stools per day. This patient did not receive any medicine that increased the risk of bleeding. Upon physical examination, the patient presented with pale skin and tenderness in the right lower abdomen without rebound pain. Haematologic examination showed anaemia, and the platelet count was within the normal range. Her coagulation time was within the normal range. No abnormalities in antinuclear antibody (ANA), p-anti-neutrophil cytoplasmic antibody (p-ANCA), ASCA or IgG4 were observed. Faecal bacterial culture, *C. difficile*, and CMV DNA were not detected. Mesenteric arteriovenous ultrasound showed no obvious abnormalities in the upper and lower mesenteric arteries or superior mesenteric vein. Contrast-enhanced abdominal CT revealed oedema caused wall thickening of the colon (Figure 1E). Colonoscopy showed diffuse oedema, erythema and bleeding ulcers from the ileocecal area to the sigmoid colon (Figures 2A,B). The terminal ileum was normal. Pathology showed that mucosal ulcers and inflammatory cells infiltrated the lamina propria. Ischaemic change was also seen in the lamina propria (Figure 2C). Based on the patient’s symptoms, imaging and colonoscopy findings, osimertinib-induced colonial bleeding and ulcers with grade 3 CTCAE was identified. Given her medical history, colon changes were diagnose drug-induced colitis.

Osimertinib was discontinued. She received prednisone 0.5 mg/kg for 1 week and the dosage was later reduced. Five days after treatment with prednisone, and lower abdominal pain was relieved. Follow-up colonoscopy showed no pathological findings. After colonoscopy, a 110 mg dose of aumolertinib, a novel third-generation EGFR-TKI used to treat patients with EGFR T790M mutation, was orally administered once daily. This patient did not experience abdominal pain or intestinal bleeding. This patient is still alive and free of colitis recurrence 5 months after aumolertinib initiation. CT re-evaluation showed stable disease (SD) in November 2021 (Figure 1F). To date, the patient has not experienced any obvious adverse events.

## Disscussion

EGFR-TKIs have become the standard first-line therapy for patients with advanced NSCLC harbouring EGFR mutations (Novello et al., 2016). Approximately 60% of patients who receive first- or second-generation EGFR-TKIs develop EGFR T790M mutation after 9–13 months of treatment (Maemondo et al., 2010). Osimertinib is recommended for patients whose previous EGFR-TKI therapy failed or for those with advanced NSCLC harbouring EGFR T790M mutation with manageable toxicity (Hanna et al., 2017; Mok et al., 2017). Previous adverse events associated with osimertinib treatment have been reported to be anaemia, diarrhoea, pulmonary disease and cardiotoxicity. Most adverse events were a Grade 1–2. Dose interruption with osimertinib was associated with diarrhoea and skin toxicity (Akamatsu et al., 2021). Osimertinib might increase bleeding risk at metastatic sites of malignancy. Diffuse alveolar haemorrhage (Forte and Sangani, 2019), subdural haemorrhage (Yu et al., 2021) and metastatic pancreatic haemorrhage (Hayasaka et al., 2018) have been previously reported. No case of intestinal haemorrhage was previously reported. For intestinal lesions, pneumatosis intestinalis induced by osimertinib was previously reported (Nukii et al., 2019). Our case report revealed severe intestinal haemorrhage and colitis as possible adverse events of osimertinib. It can be life-threatening if left undiagnosed and untreated over time. In this case, haematochezia was resolved by discontinuing osimertinib and systemic glucocorticoid treatment. Colonoscopy showed normal intestinal mucosa after treatment. Subsequent treatment with aumolertinib for NSCLC for 5 months did not show disease progress, and no haematochezia appeared.

The mechanism of osimertinib-induced colitis is unclear. Other EGFR-TKIs, such as erlotinib and gefitinib, have been reported to induce gastrointestinal bleeding without colonoscopy examination (Bai et al., 2013). EGFR is expressed in both cancer cells and healthy gastrointestinal epithelial cells. In the mammalian intestinal epithelium, EGFR is crucial for essential functions including maintenance of mucosal integrity, reducing bacterial translocation and preserving gut barrier function after injury (Van Sebille et al., 2017). After oral administration of EGFR-TKIs such as afatinib and dacomitinib, immunostaining showed strong staining was observed in the ileum and colon (Yamamoto et al., 2019a; Yamamoto et al., 2019b). Inhibition of EGFR receptors with EGFR-TKIs such as erlotinib or dacomitinib causes direct mucosal damage and altered gastrointestinal permeability (Rasmussen et al., 2010; Van Sebille et al., 2017). This may be the direct cause of colitis. In addition, immune-mediated mechanisms may also be involved. An *in vivo* study showed that gefitinib had an antiangiogenic effect (Guillamo et al., 2009), which was a potential mechanism of colonic ischaemia (Hamdeh et al., 2021). It is unclear whether osimertinib has a similar mechanism. Injury of the intestinal mucosa, drug-induced colonic ischaemia and subsequent colitis might be potential risk factors for bleeding.

In this patient, tests for *C. difficile*, CMV, EBV and stool culture were negative. She had no other risk factors for colitis. Considering her medical history and colonoscopy performance quality, she was diagnosed with drug-induced colitis. Other targeted drugs for the treatment of NSCLC, such as encorafenib and binimetinib, have been reported to induce colitis (Gelsomino et al., 2021). Drug-induced colitis presents with new-onset colitis that excludes infectious colitis and requires a history of suspicious drug use. Abdominal pain, diarrhoea and haematochezia were the most prevalent symptoms (Brechmann et al., 2019). Endoscopy findings included oedema, petechial haemorrhage, erythema, erosions or ulcers. Pancolitis is the most common presentation of luminal inflammation (Marthey et al., 2016). Histologically, there is significant overlap between drug-induced injuries and various disease entities. For the treatment of drug-induced colitis, colitis resolves with discontinued use of medication in mild cases (Hamdeh et al., 2021). Systemic glucocorticoids are a first-line therapy for moderate or severe colitis (Dougan et al., 2021). In this case, although she had received first-generation EGFR-TKI therapy, colitis did not occur until osimertinib treatment for 8 months. Due to severe colitis, osimertinib rechallenge was not appropriate. After discontinuing osimertinib and receiving glucocorticoid therapy, her symptoms improved. After treatment with aumolertinib for 5 months, the patient was in control of the disease, and no abdominal pain or haematochezia occurred. This reminded us that if patients with NSCLC harbouring EGFR T790M mutation could not tolerate the side effects of osimertinib, aumolertinib, a novel third-generation EGFR-TKI, may be a good choice.

The era of personalized medicine for advanced-stage NSCLC began with avaiable biomarkers (Camidge et al., 2019). The investigation of pathogenesis and identification of various therapeutic molecular targets has increased the significance of targeted therapy in NSCLC. EGFR-TKIs are the most widely used targeted agents and have significantly prolonged survival in EGFR-mutant patients with NSCLC (Zhang et al., 2021). EGFR-TKIs reduce the serious adverse effects of traditional chemotherapy drugs. However, diarrhoea is prominent among the side effects of targeted drugs. It can be predicted that in the next 5 years, the reports of gastrointestinal adverse reactions of EGFR-TKIs will increase mainly due to the direct mucosal damage by EGFR-TKIs. When digestive system symptoms such as abdominal pain and hematochezia occur, multidisciplinary consultation is required. We believe that with the development of pharmaceutical industry, new targeted agents with less side effects are on the way.

## Conclusion

Severe colitis is a rare adverse event associated with osimertinib. Physicians should be aware of intestinal haemorrhage associated with osimertinib. A consultation with a gastroenterologist is recommended to manage strategies to prevent adverse events of intestinal hemorrhage. Evaluation with colonoscopy and biopsy is critical for diagnosis and treatment. Discontinuation of osimertinib and treatment with corticosteroids might be effective for severe colitis. After improvement of intestinal symptoms, it might be a good choice for treating NSCLC patients with T790M mutations with aumolertinib, a novel third-generation EGFR-TKI. Aumolertinib benefited our patient and reduced the occurrence of adverse events. However, whether aumolertinib can reduce the incidence of colitis over time needs further investigation.

## Data Availability

The raw data supporting the conclusions of this article will be made available by the authors, without undue reservation.
